# Bayesian MCMC with Gibbs sampling for saturation flow rate estimation in heterogeneous traffic at pretimed signalized intersections

**DOI:** 10.1016/j.mex.2025.103507

**Published:** 2025-07-16

**Authors:** Lulusi Lulusi, Sugiarto Sugiarto, Sofyan M. Saleh, Muhammad Isya, Muhammad Rusdi, Roudhia Rahma

**Affiliations:** a Doctoral Program, School of Engineering, Post Graduate Program, Universitas Syiah Kuala, Banda Aceh, 23111, Indonesia; b Department of Civil Engineering, Universitas Syiah Kuala, Banda Aceh 23111, Indonesia; c Center for Environmental and Natural Resources Research, Universitas Syiah Kuala, Banda Aceh 23111, Indonesia; d Remote Sensing and Cartography Laboratory, Universitas Syiah Kuala, Banda Aceh, 23111, Indonesia; e Data Analitika, Terasone Saintek Nusantara, Aceh Besar 23370, Indonesia

**Keywords:** Pretimed signalized, Heterogeneous traffic, Base saturation flow rate, Bayesian, MCMC, Gibbs sampling, Pretimed signal, Mixed traffic

## Abstract

Pretimed signalized intersections significantly contribute to traffic congestion, especially under the heterogeneous traffic conditions commonly observed in emerging economies such as Indonesia. Accurate estimation of the base saturation flow rate (BSFR) is essential for reliable capacity assessment, which influences effective intersection design and operation. However, the current BSFR estimation methods outlined in the Indonesian Highway Capacity Guidelines (IHCG, 2023) rely on outdated linear models derived from the Indonesian Highway Capacity Manual (IHCM, 1997), which are inadequate for addressing contemporary heterogeneous traffic complexities. This study introduces a Bayesian Markov Chain Monte Carlo (MCMC) model employing Gibbs sampling to improve BSFR estimation accuracy. The Bayesian MCMC model achieved a Root Mean Square Error Approximation (RMSEA) of 8.638 % compared to the existing IHCG method, which produced an RMSEA of up to 51.428 %, enabling a more precise intersection capacity design. Additionally, the developed model reduced the BSFR overestimation associated with the IHCG method by approximately 42.79 %, highlighting the potential of Bayesian MCMC methods to effectively address heterogeneous traffic challenges, enhance traffic management strategies, and optimize intersection operations.

The Bayesian approach provides a probabilistic framework for quantifying uncertainty, allows for the incorporation of prior knowledge to enhance parameter estimation flexibility, and effectively mitigates model overfitting.

The developed model demonstrates robust statistical validity, characterized by a mean beta parameter value of 403.30, standard deviation of 8.66, and Monte Carlo Standard Error (MCSE) of 0.0008, confirming high reliability and predictive precision.

The proposed BSFR model exhibited superior performance in fitting empirical data, as evidenced by an RMSE of 240.403 PCU/g/h/We and RMSEA of 8.638 %, indicating an excellent model fit within acceptable thresholds (<10 %).

## Graphical abstract

**Figure:**
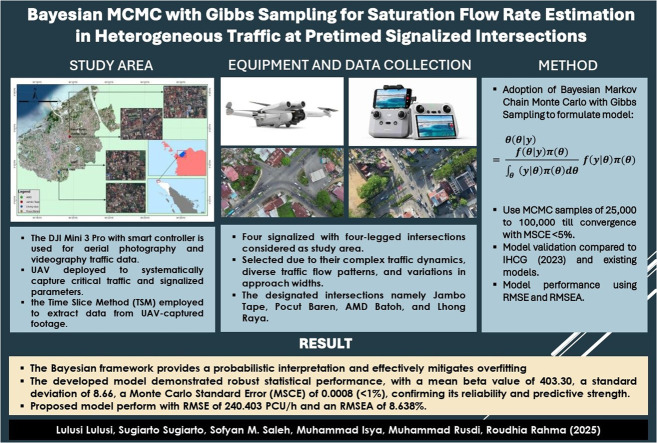

**Specifications table**

**Table:** 

**Subject area**	Engineering
**More specific subject area**	Traffic Engineering / Traffic Modelling
**Name of your method**	Bayesian MCMC
**Name and reference of original method**	S. Sugiarto, F. Apriandy, Y. Darma, S.M. Saleh, M. Rusdi, T. Miwa. Determining passenger car equivalent (PCEs) for pretimed signalized intersections with severe motorcycle composition using Bayesian linear regression, PLoS ONE 16(9): e0256620.
**Resource availability**	None

## Background

Pretimed signalized intersections serve as primary congestion points in urban areas, particularly under the heterogeneous traffic conditions prevalent in developing countries such as Indonesia. These intersections, which are characterized by fixed-phase traffic signals, often struggle to accommodate dynamically changing traffic demands. This imbalance between traffic flow and intersection capacity frequently results in excessive vehicle queuing, increased delays, and reduced overall efficiency in urban transportation networks. Given rapid urbanization and growth in vehicle ownership, managing intersection congestion has become a pressing concern for urban planners and transportation engineers [1,2], including prolonged travel times, higher vehicle operating costs, increased air pollution, and significant economic losses [3,4]. Extensive research has been devoted to developing novel methodologies in the field of traffic engineering to support the design and operation of efficient and effective transportation systems, particularly in urban areas. For instance, recent studies explored the application of fuzzy graph theory, fuzzy chromatic numbers, and fuzzy inference systems to model traffic signal control based on factors such as traffic flow, conflict points, and queue lengths at intersections [5]. Other emerging approaches include traffic modeling techniques based on centrality measures and space syntax to analyze large-scale urban road networks [6], the implementation of the YOLOv8 algorithm to train diverse Indian traffic datasets for autonomous vehicle applications [7], and the integration of urban traffic models to enhance the resilience of transportation systems [8].

A critical parameter in traffic capacity analysis is the base saturation flow rate (BSFR) that quantifies the maximum rate at which vehicles can pass through an intersection under optimal conditions. For example, efforts have been made to enhance capacity signaling by determining passenger car equivalent values for motorcycles [9] and recalibrating the BSFR [[10], [11], [12]]. BSFR significantly influences the performance of signalized intersections by directly affecting signal timing, intersection capacity, and level of service [11,13,14]. Extensive research has focused on examining the impacts of passenger car units (PCUs) and saturation flow rates in heterogeneous traffic with the aim of optimizing intersection capacity design by incorporating saturation flow and vehicle equivalency factors in motorcycle-dominated traffic [[11], [12]], and severely heterogeneous traffic conditions [[13], [14]]. Developing traffic signal control strategies that account for real-time traffic variations is critical for optimizing intersection efficiency and improving overall traffic safety [15]. Despite their importance, conventional estimation methods often fail to accurately capture the complexities of heterogeneous traffic, resulting in suboptimal intersection performances.

One of the major challenges in estimating the BSFR is the inherent heterogeneity of traffic flow in developing countries, such as Indonesia. Unlike homogeneous traffic conditions typically observed in developed nations, Indonesia's urban roads accommodate a diverse mix of vehicles, including motorcycles, passenger cars, buses, and trucks. Motorcycles constitute a significant proportion of the traffic stream and exhibit unique behavioral patterns such as lane changing and non-lane-based movements [16,17]. This irregular traffic behavior generates complex traffic flow dynamics that conventional BSFR estimation methods fail to accurately capture [18,19].

The BSFR estimation method in the Indonesian Highway Capacity Manual (IHCM) [20] and its updated version, the Indonesian Highway Capacity Guidelines [21], remains based on a simplistic linear regression model derived from traffic data collected in 1997. Despite the revisions introduced in IHCG [21], the empirical modeling framework has remained fundamentally unchanged from the IHCM [20], failing to incorporate contemporary advancements in traffic analysis methodologies. This outdated empirical model does not adequately represent the evolving traffic dynamics, which have been significantly influenced by rapid urbanization, increased vehicle ownership, and transformations in transportation infrastructure [[22], [23], [24]]. Continued reliance on these conventional models poses a considerable risk of estimation inaccuracies, thereby undermining the effectiveness of intersection signal-timing optimization and traffic management strategies. Consequently, relying on traditional models may lead to inaccurate estimations, ultimately affecting the efficiency of intersection signal timing plans and traffic management strategies.

Recent advancements in computational modeling and data analytics have opened new avenues for more sophisticated BSFR estimation approaches. Studies have demonstrated that statistical and machine learning methods can achieve superior accuracy compared with traditional linear models [11,[14], [15], [16],^19^]. These advanced techniques allow for the integration of high-dimensional traffic data and enable the modeling of nonlinear relationships among traffic parameters. Although machine learning models exhibit high predictive power, they often lack interpretability and require large datasets for training, making them less practical for real-world applications. A promising alternative to conventional regression and machine learning models is the Bayesian Markov Chain Monte Carlo (MCMC) approach, particularly with Gibbs sampling. Bayesian inference provides a probabilistic framework that allows for the integration of prior knowledge and the quantification of uncertainty in traffic parameter estimation [15,16,19]. The Bayesian MCMC approach offers several advantages, including enhanced estimation accuracy, robustness to data noise, and the capacity to handle missing or sparse data effectively [25,26]. Furthermore, the Bayesian nonparametric instrumental variable approach could mitigate the risk of overfitting by balancing model complexity with predictive accuracy, making it well suited for heterogeneous traffic conditions [27].

Numerous studies have highlighted the effectiveness of Bayesian MCMC in traffic analysis, particularly for BSFR estimation. Bayesian stochastic approaches effectively capture variability in traffic flow by accommodating complex interactions among different vehicle types [18]. Moreover, the Bayesian framework for real-time traffic estimation demonstrated its superior capability in quantifying the uncertainty in traffic data. MCMC algorithms are widely used to explore and sample complicated high-dimensional target probability distributions [28]. These studies showed that Bayesian inference provides more reliable predictions than deterministic models, particularly under highly variable traffic conditions. Similarly, the Bayesian Tobit model [15] and MCMC algorithms have been widely used to explore and sample complicated high-dimensional target probability distributions [28]. Furthermore, Bayesian MCMC methods have proven effective in traffic parameter estimation, reducing estimation bias, and improving model stability [15]. Their findings indicated that Bayesian MCMC methods perform particularly well in non-stationary traffic environments, where traditional regression models often fail to provide reliable predictions. Emphasizing the inadequacy of simple linear models [16,19] in capturing the relationship between effective approach width and BSFR, these studies suggest that probabilistic models such as Bayesian inference offer a more flexible and accurate means of modeling such relationships. Bayesian inference remained largely a theoretical construct with limited practical application until the introduction of efficient approximation techniques and Markov Chain Monte Carlo (MCMC) simulation [29,30].

Building on the extensive evidence supporting the advantages of Bayesian MCMC, this study explicitly aims to develop a Bayesian MCMC model incorporating Gibbs sampling for more accurate BSFR estimation under heterogeneous traffic conditions and to validate the model's performance by comparing it with both the IHCG and other established methods. The Bayesian MCMC model proposed in this research incorporates Gibbs sampling to enhance estimation accuracy while accounting for the inherent complexities of heterogeneous traffic environments.

This study presents a novel methodological framework to improve the accuracy of the base saturation flow rate (BSFR) estimation under heterogeneous traffic conditions. The proposed Bayesian MCMC approach incorporating Gibbs sampling demonstrated superior predictive performance compared to conventional estimation techniques. Methodologically, this study advances intersection capacity analysis by integrating prior knowledge, probabilistic reasoning, and real-time traffic data within a coherent Bayesian inference framework.

From a practical perspective, the model enables more effective traffic planning, optimized signal control strategies, and adaptive traffic management. The findings of this study will contribute to the development of more reliable, efficient, and sustainable urban transportation systems, particularly in the context of complex mixed-traffic environments. Unlike previous studies that relied on conventional regression or machine learning models, this study introduced a probabilistic Bayesian MCMC framework specifically tailored to heterogeneous traffic conditions in Indonesia. To the best of our knowledge, this is the first study to integrate Gibbs sampling within a Bayesian MCMC approach for BSFR estimation in this traffic context, addressing the limitations of existing methods and enhancing the estimation accuracy in complex mixed traffic environments. The revised section is highlighted in yellow for clarity. Notwithstanding the contributions of this study, some limitations must be acknowledged. The Bayesian MCMC model, which demonstrates enhanced accuracy and flexibility for BSFR estimation, is sensitive to the specification of prior distributions, and requires considerable computational resources to achieve convergence. Additionally, the model's dependence on UAV-based data collection introduces potential operational constraints, particularly under unfavorable weather conditions, which may limit data acquisition and quality. These methodological limitations were explicitly recognized to ensure a comprehensive and transparent evaluation of the proposed approach.

## Method details

### Study site and equipment

**Fig. 1:**
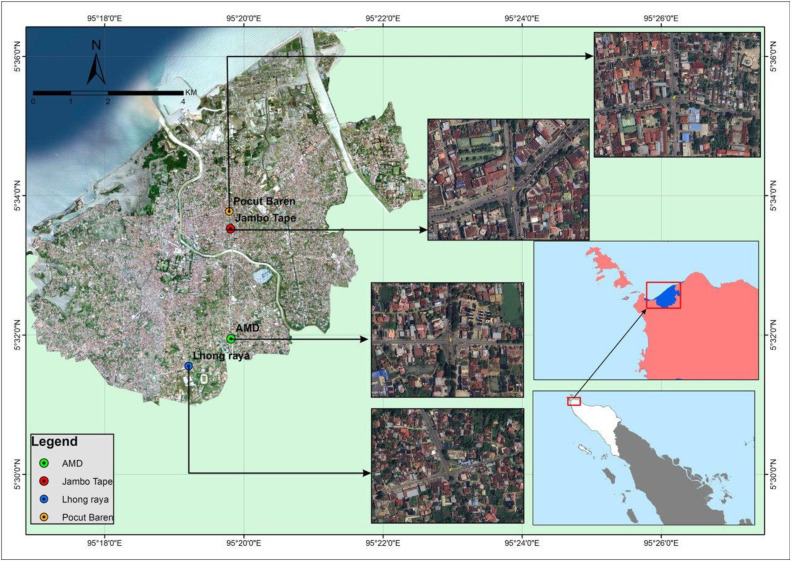
Location of study.

The selection of study locations was based on systematic observations conducted prior to the BSFR analysis and during the data-collection phase. The study sites were chosen from a range of signalized four-legged intersections that exhibited similar characteristics, to ensure consistency in comparative analysis. Consequently, four signalized four-legged intersections in Banda Aceh, the capital of the Aceh Province in the westernmost part of Indonesia, were identified as suitable study locations. These intersections were selected because of their complex traffic dynamics, diverse traffic flow patterns, and variations in approach widths. The designated intersections included Jambo Tape, Pocut Baren, AMD Batoh, and Lhong Raya (see Fig. 1).

**Fig. 2:**
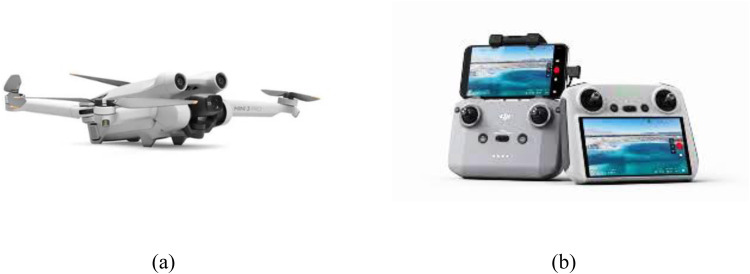
Equipment for data collection: (a) DJI 3 Mini Pro; (b) Smart Controller.

The preliminary phase of the research involved preparation of the equipment and instruments necessary for monitoring traffic characteristics, with a primary focus on data acquisition through UAV recordings. A DJI Mini 3 Pro equipped with a smart controller was used in this study, as shown in Fig. 2. DJI Mini 3 Pro is a state-of-the-art aerial imaging device capable of adapting to various lighting conditions. Equipped with a 1/1.3-inch CMOS sensor that supports dual native ISO and direct HDR output, this drone captures enhanced details in both highlights and shadows, resulting in a higher dynamic range. With larger 2.4 μm pixels and an f/1.7 aperture, the DJI Mini 3 Pro optimizes the light intake, ensuring sharp and natural images even in low-light environments. Its ability to record 4 K HDR videos and produce 48MP RAW photos makes it an ideal tool for professionals and aerial photography enthusiasts seeking superior visual quality.

### Data collection method

An unmanned aerial vehicle (UAV) was deployed to systematically capture critical traffic parameters, including vehicle classification, base saturation flow rate, and effective green time, at the analyzed intersections. The geometric characteristics of each intersection approach were directly measured on-site to ensure precision in subsequent analytical processes. Traffic flow data were meticulously collected during peak periods, encompassing the morning rush hour (06:45–08:30 AM) and evening peak (04:45–06:30 PM). The UAV was strategically positioned to obtain comprehensive aerial footage of departing traffic movements from each approach, thereby optimizing data acquisition. Owing to battery constraints of the UAV, periodic replacements were conducted every 20 min to maintain uninterrupted surveillance. Additionally, signal timing parameters were acquired through direct field observations, wherein surveyors employed a digital stopwatch to precisely document phase durations. This technique is used to measure the initial time lost at each intersection. The integration of these methodological measures ensured a high degree of accuracy and reliability in traffic flow assessment, reinforcing the robustness of the subsequent analytical framework.

**Fig. 3:**
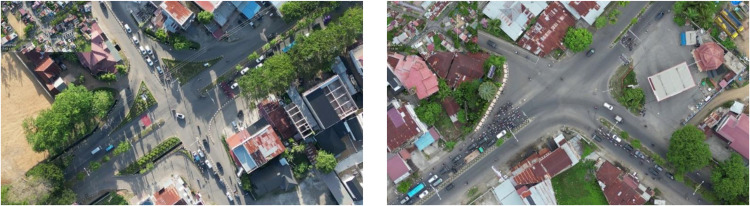
The UAV position to obtain comprehensive aerial footage of traffic data.

The core of data processing in this study was centered on the analytical evaluation of recorded video footage, which captured critical traffic parameters such as vehicle composition, base saturation flow rate, and effective green time. To ensure precision and consistency, data were collected over multiple green signal cycles for each intersection approach. The extraction process entailed systematic quantification of the number of vehicles traversing the stop line during the green phase for both through-going and right-turning movements, as illustrated in Fig. 3. In this study, the time-slice method (TSM) was employed to derive the base saturation flow data from UAV-captured footage. The TSM, also known as the Webster method, is utilized to assess vehicle flow fluctuations throughout the green phase [31]. The classified vehicle counts were obtained by segmenting the green phase duration into a series of uniform time intervals (typically 3–6 s), enabling detailed temporal analysis of traffic flow patterns. The application of the TSM facilitates a granular investigation of vehicle discharge dynamics at intersections, particularly in scenarios characterized by heterogeneous queue formations, allowing for a more refined assessment of flow consistency. To analyze signalized intersections, the details of each parameter for a lane-based car-dominated traffic stream can be considered, with limited applicability for mixed traffic conditions [32]

Following the data extraction process, the recorded vehicle composition, base saturation flow rate, and green time metrics were systematically structured into tabular data sets. The observed base saturation flow rate (BSFR) was determined by measuring the time interval from the moment the first vehicle crossed the stop line at the onset of the green signal until the last queued vehicle cleared the stop line before activating the red signal. The saturation flow data obtained through this methodology were subsequently utilized as key input variables for advanced traffic flow analysis, employing Bayesian Markov Chain Monte Carlo (MCMC) modeling techniques.

### Bayesian MCMC approach for BSFR accuracy

The variability in vehicle classifications was normalized into Passenger Car Units (PCU) using the Equivalent Passenger Car Unit (EPCU) methodology, as outlined in the Indonesian Highway Capacity Guidelines [21], thereby converting the saturation flow rate into PCU-based units. As saturation flow and effective green time on an approach to a traffic signal are very important inputs to methods of estimating delay-minimizing or capacity maximizing signal settings [33] By adopting a multiple linear regression method of estimating parameters of the traffic signal departure process [34]. Assuming a simple linear relationship between saturation flow rate and approach width, the average saturation flow rate (S₀) and effective lane width were systematically analyzed to formulate a predictive model for saturation flow rate (S₀). Notably, the model formulated in this study was intentionally designed to be straightforward, ensuring its suitability for comparative analysis with the saturation flow rate model in IHCG 2023 [21]. This methodological alignment adheres to the principle of simplicity, which is fundamental to the baseline model outlined in the manual in which the effective lane width is the sole independent variable considered in the analysis.

The Bayesian method, as referenced in a previous study by [16], was applied in this study to calibrate Passenger Car Equivalents (PCE) using a Bayesian inference approach. Specifically, Bayesian modeling was used to estimate the Base Saturation Flow Rate (BSFR) through a linear regression framework. The BSFR formulation assumes that an experimental dataset consists of exogenous variables, denoted by *Y* = {Yi}, which correspond to distinct values of the independent variable X. Furthermore, stochastic experiments indicate that multiple values of Yi may exist for the same level of Xi. Under the assumption that the probability distribution [fi (Y|X)] maintains a constant variance (σ2) across all values of X, the relationship between response and predictor variables is expected to follow a linear pattern, forming what is referred to as the true regression line. The expected mean response is defined as μi = *E* (Yi), as shown in Equation (3). Additionally, the population parameters (α and β) governing the regression line were estimated from the observed dataset, following the mathematical formulation provided in Eq. (1).

(1) $$E\left(Y_{i}\right)=\alpha +\beta X_{i}$$

Parameter coefficients α and β were calibrated using the observed dataset obtained from the survey. The Ordinary Least Squares (OLS) method is a widely used approach to this calibration. In the subsequent step, Bayesian inference is applied to estimate the regression parameters within the OLS framework by assuming a predefined prior distribution for the parameters. The prior distribution (πθ) represents prior knowledge regarding the dataset being analyzed. To refine this prior belief, a likelihood function is utilized, which updates the prior distribution, thereby obtaining the posterior distribution (π(θ|y)) of the parameters. This posterior distribution provides a probabilistic estimate of the regression parameters by incorporating both prior assumptions and observed data. Based on the fundamental assumptions governing the Bayesian regression framework, the posterior distribution of θ as formulated as:

(2) $$\theta \left(\theta |y\right)=\frac{f\left(\theta |y\right)\pi \left(\theta \right)}{\int_{\theta }\left(y|\theta \right)\pi \left(\theta \right)d\theta }\;\propto f\left(y|\theta \right)\pi \left(\theta \right)$$

In this Bayesian framework, the observed outcomes are represented by *y* = {y_1_,…,y_2_,…y_n_}, where yi corresponds to an empirical data point. The sampling distribution is denoted by π(y|θ), which describes the likelihood of the observed data given the model parameters. Additionally, the marginal distribution of y is expressed as $\int_{\theta }\left(y|\theta \right)\pi \left(\theta \right)d\theta$ which integrates over all possible values of θ, incorporating both the likelihood function and the prior distribution.

The superior performance of the Bayesian model over linear and exponential models has been well-documented in the literature. Bayesian inference is known for its ability to incorporate prior knowledge and update parameter estimates iteratively, leading to improved accuracy and robustness in predictive modeling [35]. Compared to traditional ordinary least squares (OLS) regression, which assumes fixed parameters and relies solely on available data, Bayesian modeling accounts for parameter uncertainty and provides probability distributions rather than point estimates, allowing for better adaptability to real-world variations [36]. Furthermore, assume a fixed growth rate of ordinary least squares, which may not adequately capture the stochastic nature of the observed data [37]. Bayesian hierarchical models further confirm that they outperform traditional regression models by reducing overfitting and improving generalization across diverse traffic conditions [[38], [39], [40]].

Bayesian inference in this study is conducted using Gibbs sampling, a Markov Chain Monte Carlo (MCMC) algorithm that sequentially samples from the conditional distributions of the parameters. The model parameters were calibrated using Python (https://www.python.org) to enable an efficient computational analysis and optimization. The predictive model calibration for the intersection saturation flow rate was based on empirical data obtained from four signalized intersections encompassing 16 intersection approaches. The developed model was subsequently validated by comparing its predicted saturation flow rates with the observed values and benchmarking the results against [21] model to evaluate its accuracy and reliability. Furthermore, the convergence of the Bayesian model uses the Monte Carlo Standard Error (MSCE), ensuring that the estimated standard deviation remains below 0.05, as recommended in [16,25,26]. The Bayesian Implementation Process was conducted using the following steps:

1) Extraction of Traffic Parameters: Cycle time data, signal phase information, and saturation flow values were obtained from UAV-based traffic monitoring data and processed for further analysis.
2) Adoption of Bayesian Linear Modeling: The Bayesian modeling framework was implemented using Python, ensuring computational efficiency and reproducibility.
3) Bayesian Inference with Gibbs Sampling implemented in accordance with Eqs. (1) and (2), the Bayesian analysis then conducted using the following parameters: (a) MCMC samples = 15,000; (b) MCMC samples = 25,000; (c) MCMC samples = 50,000; and (d) MCMC samples = 100,000.
4) Posterior Parameter Estimation and Convergence Check: The posterior estimates of the model parameters were assessed using the Monte Carlo Standard Error (MSCE) to verify the convergence threshold criterion set at ≤ 5 %.
5) Model Calibration and Validation: Calibrated BSFR using Bayesian MCMC and validated by comparing models’ performance to: (a) IHCG Model (2023); (b) Munawar (2005); (c) Fazila et al. (2024); and (d) observed BSFR data.
6) To evaluate the effectiveness of the model, Root Mean Square Error (RMSE) and Root Mean Square Error Approximation (RMSEA) were computed to provide a quantitative visualization of predictive accuracy.

### Empirical result interpretation

To calibrate the BSFR model, 480 cycles of observed BSFR data, comprising four pre-timed signal intersections and 16 approaches/legs, were used. The aggregate value of the observed BSFR is used for each approach/leg. Therefore, the calibration of the BSFR model used 16 observed BSFR and their corresponding effective widths (We). In this simple linear regression, the dependent variable is only regressed to the exogenous variable of effective width (We) as recommended by [21]. The purpose of using a simple linear model is to validate the existing BSFR formula by IHCG [21] and verify whether it can still be used for the design and operation of signalized intersections particularly in the study area.

The BSFR model, based on a simple linear formulation, was estimated using Bayesian analysis with regression conducted through Gibbs sampling within the MCMC framework across multiple iteration stages (25,000, 50,000, 75,000, and 100,000 iterations). Table 1 presents the Bayesian estimation results for the effective width (We) of this approach. The findings indicate that as the number of iterations increases from 25,000 to 100,000, the mean parameter estimates remain stable, ranging from 403.15 403.30. This stability confirms that the estimation process achieves convergence, thereby ensuring the reliability of the results. Additionally, the standard deviation (SD) values, ranging from 8.61 to 8.69, demonstrate minimal and consistent variability in the parameter estimates across all iteration stages.

**Table 1:** -.

Parameters	MCMC Iterations	Mean	SD (Standard Deviation)	MSCE (Monte Carlo Standard Error)
Beta (We)	25,000	403.15	8.62	0.0033
Beta (We)	50,000	403.23	8.61	0.0016
Beta (We)	75,000	403.25	8.69	0.0011
Beta (We)	100,000	403.30	8.66	0.0008

The goodness of fit of the model was further assessed using the Monte Carlo Standard Error (MCSE), as presented in Table 1. The MCSE values decreased substantially, from 0.0033 at 25,000 iterations to 0.0008 at 100,000 iterations, indicating a significant reduction in the estimation uncertainty. These results clearly demonstrate that the model achieves optimal stability at 100,000 iterations when the parameter estimates are both precise and reliable. In practical terms, this suggests that further increasing the number of iterations beyond 100,000 is unlikely to yield substantial improvements, thus confirming the computational efficiency of the model at this stage. The combination of stable parameter estimates, low MCSE values, and minimal variation in SD indicates that the proposed Bayesian model provides a good fit to the observed data, thereby capturing the underlying traffic flow dynamics with high precision.

The empirical relationship derived from the proposed model indicates that BSFR = 403.30We, in contrast to the existing IHCG (2023) formulation that uses BSFR = 600We. This discrepancy highlights a critical issue with the IHCG (2023) model, suggesting that it tends to overestimate the BSFR, particularly as the effective approach width (We) increases. Such overestimation can lead to unrealistic intersection capacity predictions, potentially undermining the traffic signal design and operational efficiency. Consequently, these findings underscore the need to recalibrate the BSFR formula within the IHCG (2023) model to reflect heterogeneous traffic conditions more accurately. Importantly, the superiority of the proposed Bayesian model lies not only in its improved estimation accuracy, but also in its ability to account for traffic composition and variability of factors often overlooked in conventional approaches. Refining the BSFR model based on updated empirical data enhances the reliability of the capacity estimations for signalized intersections, ultimately supporting more effective traffic management and infrastructure planning.

### BSFR model validation

Table 2 shows the validation of the proposed model and presents a comparative analysis between the observed BSFR (PCU/g/h/We) and four predictive models: Proposed Bayesian Model (BSFR = 403.30 We), IHCG 2023 (BSFR = 600 We), Exponential Model (BSFR = 500 We^0.95, Munawar 2005), and OLS Model (BSFR = 350 We, Fazila et al. 2024). Based on the computed Root Mean Square Error (RMSE) and Root Mean Square Error Approximation (RMSEA), the Proposed Bayesian Model demonstrated the best performance in representing the observed data, with an RMSE of 240.403 PCU/g/h/We and an RMSEA of 8.638 %. In contrast, the IHCG 2023 model exhibited the highest error, with an RMSE of 1431.183 PCU/g/h/We and an RMSEA of 51.428 %, indicating its lower accuracy in predicting the BSFR compared to the other models. The Exponential Model (Munawar 2005) and OLS Model (Fazila et al. 2024) showed higher errors than the Bayesian model, with RMSE values of 430.136 PCU/g/h/We and 451.699 PCU/g/h/We, respectively, and RMSEA percentages of 15.456 % and 16.231 %, respectively.

**Table 2:** Validation proposed model.

Approach Width (We) (m)	Observed BSFR (PCU/g/h/We)	Proposed Bayesian (BSFR=403.30*We) (PCU/g/h/We)	IHCG 2023 (BSFR=600*We) (PCU/g/h/We)	Exponential (BSFR=500*We^0.95 (Munawar 2005)) (PCU/g/h/We)	OLS (BSFR=350*We (Fazila et al. 2024)) (PCU/g/h/We)
3.46	1558	1399	2076	1626	1211
3.94	1858	1593	2364	1839	1379
5.00	1877	2022	3000	2307	1750
5.65	1885	2284	3390	2591	1978
6.00	1893	2426	3600	2743	2100
6.10	2230	2466	3660	2786	2135
6.58	2453	2660	3948	2994	2303
6.70	2404	2709	4020	3046	2345
6.80	2988	2749	4080	3089	2380
7.06	3064	2854	4236	3201	2471
8.20	3464	3315	4920	3691	2870
8.35	3523	3376	5010	3755	2923
8.85	3658	3578	5310	3968	3098
9.15	3753	3699	5490	4096	3203
9.50	3945	3841	5700	4244	3325
9.60	3973	3881	5760	4287	3360
RMSE (PCU/g/h/We)		240.403	1431.183	430.136	451.699
RMSEA ( %)		8.638	51.428	15.456	16.231

The comparative validation presented in Table 2 indicates that the proposed model provides the best approximation of the observed BSFR values, highlighting its reliability and practical applicability in heterogeneous traffic environments. In contrast, the IHCG (2023) model exhibited the highest estimation error, with an RMSE of 1431.183 PCU/g/h/We and RMSEA of 51.428 %. This substantial overestimation confirms that the existing IHCG (2023) formula derived from outdated traffic data fails to adequately capture the complexities of modern heterogeneous traffic systems. Similar overestimation concerns have been raised in previous studies, which emphasize the limitations of applying traditional regression-based models to diverse traffic conditions [12,13]. The Exponential Model by Munawar (2005) and OLS Model by Fazila et al. (2024) provided improved accuracy compared to the IHCG model, yet their RMSE and RMSEA values (430.136–451.699 PCU/g/h/We and 15.456 %–16.231 %, respectively) still exceeded those of the Bayesian model. These findings underscore the ability of the Bayesian approach to reduce overfitting and improve generalization across varying traffic conditions, which is a key limitation of conventional linear and exponential models. Moreover, the proposed model outperforms traditional regression models by reducing overfitting and improving generalization across diverse traffic conditions. These findings align with the results in Table 2, where the Bayesian model demonstrated lower RMSE and RMSEA, indicating a superior fit to the observed BSFR data. The Bayesian approach provides a more reliable and robust estimation framework by dynamically updating the parameter estimates and accounting for uncertainty. Consequently, this model is a more effective tool for BSFR estimation based on approach width, offering improved precision and predictive reliability compared to conventional linear and exponential models.

**Fig. 4:**
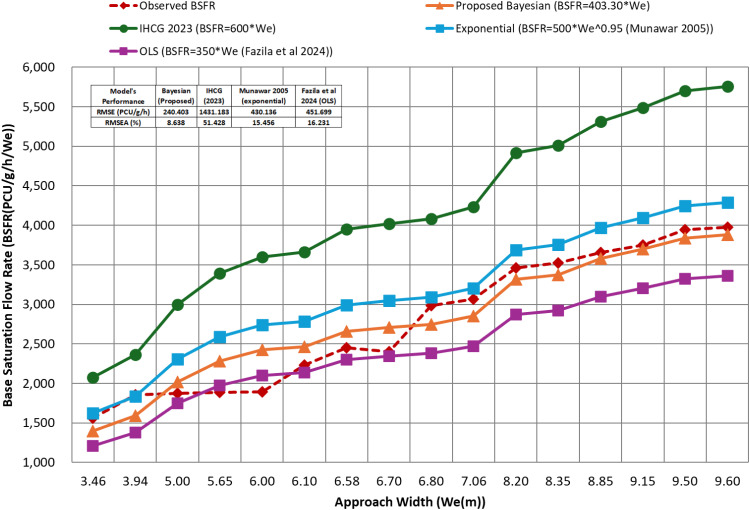
Validation proposed model.

Fig. 4 illustrates the comparison between the observed Base Saturation Flow Rate (BSFR) and various predictive models, including the Proposed Bayesian Model, IHCG 2023, Exponential Model, and OLS Model, in relation to the Approach Width (We). The visualization indicates that the Proposed Bayesian Model (BSFR = 403.30 We) closely aligns with the observed data, demonstrating superior accuracy compared to the other models, as supported by its lowest RMSE of 240.403 PCU/g/h/We and RMSEA of 8.638 %. In contrast, the IHCG 2023 model (BSFR = 600We) consistently overestimated the BSFR values relative to observations and other models, further reinforced by its highest RMSE of 1431.183 PCU/g/h/We and RMSEA of 51.428 %, indicating a significant lack-of-fit. Moreover, The Exponential Model (BSFR = 500We^0.95, Munawar 2005) and the OLS Model (BSFR = 350We, Fazila et al. 2024) provide better estimates than IHCG 2023 but still exhibit greater errors compared to the Proposed Bayesian Model, with RMSE values of 430.136 PCU/g/h/We and 451.699 PCU/g/h/We, and RMSEA percentages of 15.456 % and 16.231 %, respectively.

The visual comparison of model predictions with observed data further reinforces these conclusions, with the proposed Bayesian model consistently aligning closely with actual observations, whereas the IHCG (2023) model systematically overestimated the BSFR. These results highlight the practical implications of adopting a Bayesian model to support more accurate capacity estimation, intersection design, and traffic management strategies. However, certain limitations of this study must be acknowledged. The Bayesian model requires careful selection of prior distributions and sufficient computational resources to ensure model convergence, which may limit its operational scalability in real-time traffic management applications.

## Conclusions

This study confirms that the Proposed Bayesian Model offers the most accurate and reliable estimation of the Base Saturation Flow Rate (BSFR) based on the Approach Width (We), outperforming the traditional linear and exponential models. Its ability to converge to stable parameter estimates while minimizing errors highlights its superiority in predictive modeling. A lower RMSE (240.403 PCU/g/h/We) and RMSEA (8.638 %) validated its better fit to the observed data, reinforcing its relevance in transportation research. The comparative validation further demonstrated the effectiveness of the Bayesian model. The IHCG 2023 model significantly overestimated the BSFR, producing the highest RMSE (1431.183 PCU/g/h/We) and RMSEA (51.428 %), whereas the Exponential and OLS models still showed greater errors, although slightly improved. These findings align with prior research, confirming that Bayesian hierarchical models outperform conventional regression approaches by accounting for uncertainty and offering probabilistic estimates.

This study highlights the need to recalibrate the BSFR formula in the IHCG (2023) model to enhance prediction accuracy and improve signalized intersection capacity estimation. The discrepancy between the proposed and existing models suggests that the IHCG (2023) formula may not fully capture variations in traffic composition and operational conditions in heterogeneous traffic environments. Updating empirical data and incorporating specific traffic characteristics can improve the accuracy and applicability of a model. Furthermore, the current study was based on traffic data collected exclusively from selected intersections in Banda Aceh, Indonesia. Therefore, further research is required to evaluate the transferability of the model to other urban contexts with different traffic compositions and geometric characteristics.

## Limitations

Not applicable.

## Ethics statements

Not applicable.

## CRediT authorship contribution statement

**Lulusi Lulusi:** Conceptualization, Software, Data curation, Writing – original draft. **Sugiarto Sugiarto:** Methodology, Investigation, Formal analysis, Writing – review & editing. **Sofyan M. Saleh:** Validation, Funding acquisition, Resources, Supervision. **Muhammad Isya:** Funding acquisition, Resources, Supervision. **Muhammad Rusdi:** Software, Data curation, Visualization. **Roudhia Rahma:** Data curation, Software, Validation.

## Declaration of competing interest

The authors declare that they have no known competing financial interests or personal relationships that could have influenced the work reported in this study.

## Data Availability

Data will be made available on request.
